# Supplementary material for: Genomic and functional characterization of bacteriocinogenic lactic acid bacteria isolated from Boza, a traditional cereal-based beverage
Source: Sci Rep. 2022 Jan 27;12:1460. doi: 10.1038/s41598-022-05086-1 (PMC8795150; doi:10.1038/s41598-022-05086-1)
Supplement: Supplementary file 5 — Supplementary Information. [file 41598_2022_5086_MOESM5_ESM.pdf]

Supplementary informations

Genomic and functional characterization of bacteriocinogenic lactic acid bacteria isolated from Boza, a traditional cereal-based beverage

Luciano Lopes Queiroz[1,$], Christian Hoffmann[2,$,*], Gustavo Augusto Lacorte[2,3], Bernadette Dora Gombossy de Melo Franco[2], Svetoslav Dimitrov Todorov[4,#,*]

1. Microbiology Graduate Program, Department of Microbiology, Institute of Biomedical Science, University of São Paulo, São Paulo, SP, Brazil

2. Food Research Center (FoRC), Universidade de São Paulo, Faculdade de Ciências Farmacêuticas, São Paulo (SP), Brazil

3. Instituto Federal de Minas Gerais, Bambuí (MG), Brazil

4. Laboratório de Microbiologia de Alimentos, Departamento de Alimentos e Nutrição Experimental, Faculdade de Ciências Farmacêuticas, Universidade de São Paulo, São Paulo (SP), Brazil

Supplementary Methods

Antibiotic susceptibility

Selected antibiotics (Antibiotic discs, Oxoid Basingstoke, Hampshire, UK) for susceptibility test were used to assess interaction of the selected strains with commercial antimicrobials of different modes of action (listed in Supplementary Table 6). Studied strains was cultured in MRS broth at 37 °C for 24 h and MRS agar plates were inoculated with around 10^6-10^7 CFU/ml. The antibiotic discs were placed on the surface of a plate and subsequently incubated at 37 °C for 24 h. Inhibition zones around the discs were recorded (mm) and results used to determine the strain sensitivity[1]. The test was performed in triplicate.

Supplementary Figures

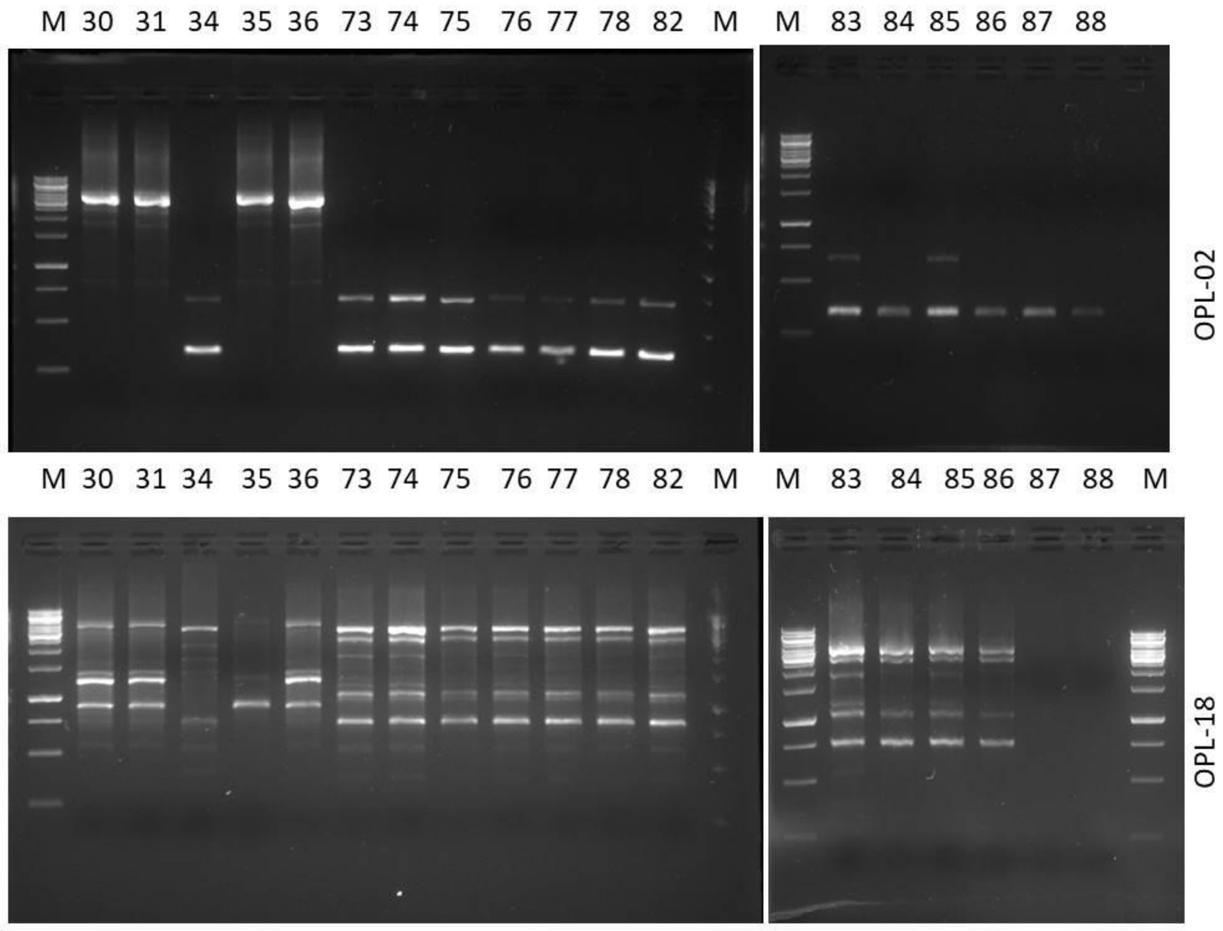

Supplementary Figure 1. RAPD-PCR profile of the 18 bacterial isolates (numbers of the isolates are indicated on the figure) with antimicrobial activity, obtained in the screening process for new bacteriocinogenic strains isolated from boza with primer OPL-02 and OPL-18. M: 1 kb ladder

(Thermo Fisher Scientific). Isolates 31, 75 and 87 were selected for further studies based on the obtained profile and recorded bacteriocinogenic activity, and identified as *Pediococcus acidilactici* ST31BZ, *Pediococcus pentosaceus* ST75BZ and *Pediococcus pentosaceus* ST87BZ, respectively.

Supplementary Tables

Supplementary tables 1, 2, 3, and 4 are available in separate files.

Supplementary Table 5. Bacteriocin susceptibility assay results, including species name and number of strains tested.

Test organisms	growth media	Bacteriocin activity sensitive strains / total number tested strains		
		ST31BZ	ST75BZ	ST87BZ
Enterococcus faecalis	MRS	14 / 20	12 / 20	12 / 20
Enterococcus faecium	MRS	12 / 13	10 / 13	10 / 13
Enterococcus hirae	MRS	3 / 3	3 / 3	3 / 3
Lactococcus lactis	MRS	8 / 11	6 / 11	8 / 11
Listeria innocua	BHI	5 / 5	5 / 5	5 / 5
Listeria monocytogenes	BHI	36 / 38	37 / 38	37 / 38
Streptococcus thermophilus	MRS	6 / 11	4 / 11	4 / 11
Latilactobacillus sakei	MRS	4 / 7	4 / 7	4 / 7
Lactiplantibacillus plantarum	MRS	0 / 18	0 / 18	0 / 18
Limosilactobacillus fermentum	MRS	0 / 7	0 / 7	0 / 7
Streptococcus infantarius subsp. *infantarius*	MRS	0 / 2	0 / 2	0 / 2
Limosilactobacillus mucosae	MRS	0 / 2	0 / 2	0 / 2
Salmonella spp.	BHI	0 / 8	0 / 8	0 / 8
Staphylococcus aureus	BHI	0 / 14	0 / 14	0 / 14
Staphylococcus epidermidis	BHI	0 / 4	0 / 4	0 / 4
Leuconostoc mesenteroides	MRS	0 / 9	0 / 9	0 / 9
Enterococcus mundtii	MRS	0 / 1	0 / 1	0 / 1
Latilactobacillus curvatus	MRS	0 / 3	0 / 3	0 / 3
Pediococcus spp.	MRS	0 / 6	0 / 6	0 / 6
Lactobacillus delbrueckii	MRS	0 / 2	0 / /2	0 / 2

Supplementary Table 6. Antibiotic susceptibility of *Pediococcus acidilactici* ST31BZ, *Pediococcus pentosaceus* ST75BZ and *Pediococcus pentosaceus* ST87BZ.

Antibiotic	μg/disk	ST31BZ	ST78BZ	ST85BZ
		Diameter inhibition zone (mm)		
chloramphenicol	30	25	22	25
neomycin	10	18	16	16
ampicilin	10	0	0	0
tobramycin	10	0	0	0
kanamycin	30	0	0	0
gentamycin	10	0	0	0
amikacin	30	0	0	0
oxacillin	1	0	0	0
vancomycin	30	0	0	10
bacitracin	10	17	12	12
ciprofloxacin	5	0	0	0
ceforoxime	30	26	22	23
imipenem	10	28	28	29
clindamycin	2	25	24	24
erytromycin	15	23	22	23
cefepime	30	18	16	15
penicillin	10	27	22	22
tetracicline	30	0	0	0
metronidazole	50	0	0	0
nalidix acid	30	0	0	0

Supplementary References

1. Charteris, W. P., Kelly, P. M., Morelli, L. & Collins, J. K. Gradient Diffusion Antibiotic Susceptibility Testing of Potentially Probiotic Lactobacilli. *J. Food Prot.* **64**, 2007–2014 (2001).
